# A comparative dosimetric study for treating left-sided breast cancer for small breast size using five different radiotherapy techniques: conventional tangential field, filed-in-filed, Tangential-IMRT, Multi-beam IMRT and VMAT

**DOI:** 10.1186/1748-717X-8-89

**Published:** 2013-04-15

**Authors:** Guang-Hua Jin, Li-Xin Chen, Xiao-Wu Deng, Xiao-Wei Liu, Ying Huang, Xiao-Bo Huang

**Affiliations:** 1 State Key Laboratory of Oncology in South China and Department of Radiation Oncology, Sun Yat-sen University Cancer Center, Guangzhou, Guangdong, 510060, PR China; 2 School of Physics and engineering, Sun Yat-sen University, Guangzhou, Guangdong, 510275, PR China

**Keywords:** Left sided breast cancer, IMRT, Dosimetry, DVH

## Abstract

**Background and purposes:**

To compare the dosimetry for the left-sided breast cancer treatment using five different radiotherapy techniques.

**Materials and methods:**

Twenty patients with left sided breast cancer were treated with conservative surgery followed by radiotherapy. They were planned using five different radiotherapy techniques, including: 1) conventional tangential wedge-based fields (TW); 2) field-in-field (FIF) technique; 3) tangential inverse planning intensity-modulated radiation therapy (T-IMRT); 4) multi-field IMRT (M-IMRT); and 5) volumetric modulated arc therapy (VMAT). The CTV, PTV and OARs including the heart, the regions of coronary artery (CA), the contralateral breast, the left and right lung were delineated. The PTV dose was prescribed 50Gy and V_47.5_≥95%. Same dose constraint was used for all five plans. The planned volumetric dose of PTV and PRV-OARs were compared and analyzed.

**Results:**

Except VMAT (Average V_47.5_ was 94.72%±1.2%), all the other four plans were able to meet the V95% (V_47.5_) requirement. T-IMRT plan improved the PTV dose homogeneity index (HI) by 0.02 and 0.03 when compared to TW plan and VMAT plan, and decreased the V_5_, V_10_ and V_20_ of all PRV-OARs. However, the high dose volume (≥ 30Gy) of the PRV-OARs in T-IMRT plan had no statistically significant difference compared with the other two inverse plans. In all five plans, the dose volume of coronary artery area showed a strong correlation to the dose volume of the heart (the correlation coefficients were 0.993, 0.996, 1.000, 0.995 and 0.986 respectively).

**Conclusion:**

Compared to other techniques, the T-IMRT technology reduced radiation dose exposure to normal tissues and maintained reasonable target homogeneity, VMAT is not recommended for left-sided breast cancer treatment. In five techniques, the dose-volume histogram (DVH) of the heart can be used to predict the dose-volume histogram (DVH) of the coronary artery.

## Introduction

Many studies on comparison of dose distribution for breast cancer radiotherapy techniques have been reported [1-5]. In these studies, the comparative irradiation techniques mainly include [2-6]: 1) conventional tangential wedge fields (TW), 2) field-in-field (FIF), 3) tangential fields inverse intensity-modulated radiation therapy (T-IMRT), 4) multi-field IMRT (M-IMRT) and 5) irregular surface compensator (ISC). Recently, a new technique known as volumetric modulated arc therapy (VMAT) has been introduced. Compared to the traditional forward planning, the inverse-planned modulated irradiation therapy may benefit in better target dose homogeneity index (HI) and PRV-OARs dose reduction [5,6].

The technologies mentioned above have been implemented in many institutions in China [7,8]. Although the T-IMRT is reported having better target dose homogeneity and sparing normal tissue such as the heart and the ipsilateral lung, there are still some aspects of concern. Firstly, the planning target volume (PTV) of Chinese patients, which maximum and mean volume of 589.77cc and 427.2 cc reported by Huang [9] are obviously smaller than the Caucasians one with the maximum and the mean volume of 2170 cc and 994 cc as reported by Popescu [10]. This may lead to different results in using various irradiating techniques. Secondly, for irradiation of the left breast, cardiac dose is one of the most important issues. The most serious radiation induced complication of the heart is coronary artery injury [11]. Currently reported literature mainly [12-15] focused on the volumetric dose of the heart, but few studies concentrated on the coronary artery region specifically. Xu et al [15] conjectured the cardiac dose might be associated with the breast volume for whole left breast irradiation. In their report IMRT treatment could significantly reduce cardiac dose for those clinical target volume (CTV) larger than 500 cc compared with conventional tangential techniques. In our study, we specifically compared the coronary artery dose of various radiation treatment techniques for the Chinese patient which having relative smaller PTV. Moreover, Popescu CC et al [16] reported that VMAT was able to improve dosimetry and reduce treatment time compared to conventional intensity modulated radio- therapy for loco-regional radiotherapy of left-sided breast cancer and internal mammary nodes. Whether VMAT offers dose benefits for whole left breast irradiation is another issue of our interest.

In this study, it is aimed to give some advice about the individual irradiation therapy to the patients after left conservative surgery whose planning target volume was relative smaller based on the dose comparison of five radiation methods and the irradiated dose analysis of planning target volume and OARs.

## Material and methods

Twenty patients with left-sided breast cancer were randomly selected for this treatment planning study. They have undergone breast-conserving surgery.

### Target and normal tissue delineation

**Figure 1:**
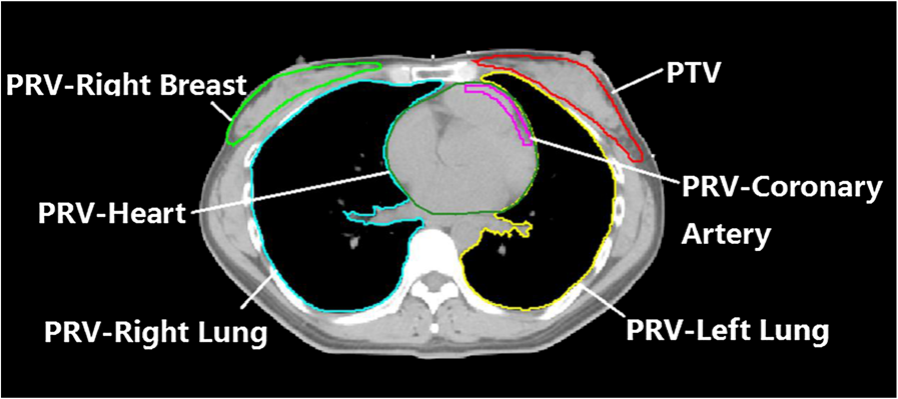
An example of the contour of PTV and PRV-OARs.

CTV and PTV for the breast were delineated according to the recommendation of ICRU report #83. The breast CTV included all visible breast parenchyma. The PTV was added a 7-mm expansion in all direction around the CTV except the skin surface, including the set-up margin and patient movement. The CTV of all the 20 cases were delineated by the same radiation oncologist based on CT image. The maximum volume of PTV was 586.4 cc, the smallest was 132.6 cc and the average was 360.8±149.1 cc. The PRV contours of all the involved OARs, including contra-lateral breast, entire heart, coronary artery area (CA), left lung and right lung were outlined by the treating physician. All targets and PRVs were outlined slice by slice of the CT image in the treatment planning system and then reconstructed the three dimensional contour automatically. Figure 1 shows the PTV and PRV-OARs.

The coronary artery most commonly affected by radiation is the left anterior descending, followed by the right branch and left circumflex [17]. Thus, the area of left front one-fourth heart 1cm subsurface can be identified as the volume of coronary artery part according to the American Memorial Sloan-Kattering cancer research methods [3].

### Plan design

All plans were completed in three-dimensional treatment planning system (Pinnacle 9.0 m, ADAC, Philips). The TPS determined homogeneous media and density in the body based on the CT density calibration curve and calculated dose with Collapse Cone convolution, which taken account of the calibration of inhomogeneous medium [18]. The Elekta Synergy linear accelerator with 6MV photon energy was used. The PTV was prescribed to 50Gy (D50%) and the optimization constraint is that ensuring 95% isodose line encompasses 95% of PTV (V95%≥47.5Gy).

The TW plan used two opposite half beam which had an appropriate wedge angle and included the whole PTV. The FIF plan and T-IMRT plan were created with same beam angle of the conventional TW plan. The FIF plan had 3–5 subfields on each side using the multileaf collimators to ensure the D_max_ of PTV not more than 52.5Gy.

As 7-beam or 9-beam plan was reported to be more appropriate for M-IMRT [19], the 7-beam plan which avoided direct exposure to the contralateral breast was selected in this study.

VMAT which arc direction is such that beam enters the breast before exiting through the lung may increase the dose volume of the lung and contralateral breast. For example, in our peer study, we found that VMAT with a partial arc could reduce the lower dose (≤10) volume of left lung nearly to 5% compared to VMAT with a full arc. So in this paper, the VMAT plan used an arc field which starting angle and ending angle were respectively the same as the tangential beam angle, and the degree of the sub-field interval of 4° was used [20].

For the IMRT and VMAT plans, the optimization objective listed in Table 1 was used. Direct machine parameter optimization (DMPO) was applied to optimize plans. The minimum field size and monitor unite of sub-field was restricted as 2 cm^2^ and 2 MU.

**Table 1:** The optimization objective used for inverse IMRT planning

**Structure**	**Planning aim**
PTV	V_52Gy_ ≤1%, V_51Gy_ ≤ 4%; D_50%_ = 50Gy; V_49Gy_ ≥ 100%,V_50Gy_ ≥ 95%
PRV-contralateral breast	D_max_ ≤ 3Gy
PRV-left lung	V_10Gy_ ≤ 30%; V_20Gy_ ≤ 20%; V_30Gy_ ≤ 10%
PRV-coronary artery region	V_10Gy_ ≤ 25%, V_20Gy_ ≤ 15%, V_30Gy_ ≤ 5%
PRV-heart	V_10Gy_ ≤ 20%; V_20Gy_ ≤ 15%; V_30Gy_ ≤ 20%

### Ethical considerations

The different treatment techniques have been applied to the patients’ dataset without any clinical application. This activity does not require an ethical approval according to our institution’s rules.

### Data analysis

The conformity index (CI) and homogeneity index (HI) were defined to describe the quality of plans as follows: 1) CI=V_47.5Gy_/PTV, V_47.5Gy_ represent the volume receiving 47.5Gy, 2) HI= (D_2%_ -D_98%_)/D_50%_, D_2%_, D_50%_ and D_98%_ mean the doses of 2%, 50% and 98% volume of the PTV.

The results difference between any two of the five plans were compared and analyzed with ANOVA test (α=0.05) using SPSS 17.0 software.

### Planned dose results

As showed in Table 2, except the VMAT plan, all the other four plans were able to meet the PTV dose prescription of V95%≥47.5Gy. The 7-IMRT had the best CI (1.3). FIF, 7-IMRT and VMAT plans had the smallest HI (0.11).

**Table 2:** **The PTV dose parameters of five plans**$\left(\bar{x}\pm d\right)$

**Parameters**	**TW**	**FIF**	**T-IMRT**	**7-IMRT**	**VMAT**
D98(Gy)	47.3±0.4	47.0±0.4	47.0±0.6	47.3±0.6	46.4±0.6
D2(Gy)	53.2±0.6	52.0±0.6	52.7±0.6	52.4±0.5	53.4±0.7
D50(Gy)	50.6±0.6	50.7±0.4	50.7±0.4	50.4±0.4	51.0±0.4
V95%	96.2±1.6^A^	95.6±1.6^A^	96.8±1.7^A^	96.1±1.7^A^	94.7±1.2^A^
CI	2.0±0.5^Aa^	1.7±0.4^A^	1.6±0.3^Ab^	1.3±0.1^Bb^	1.4 ±0.2^Bb^
HI	0.13±0.02^A^	0.11±0.02^B^	0.11±0.03^B^	0.11±0.02^B^	0.14±0.02^A^

A had significant difference with B (p<0.05), and, a had significant difference with b (p<0.05). Otherwise the difference was not significant between any two (p>0.05).

### Dose of planning target volume (PTV)

**Figure 2:**
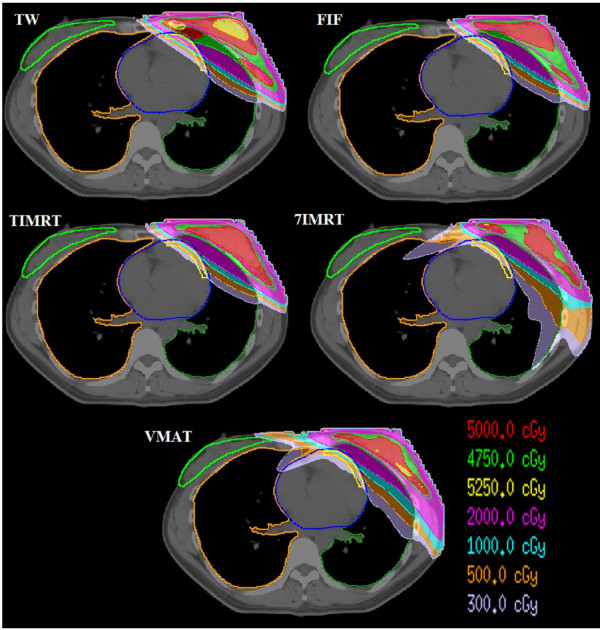
The dose distribution of five plans in isocenter slice.

The PTV_47.5_ of VMAT could not meet the planned dose constraint. In ANOVA comparison with each other plan, The CI of 7-IMRT and VMAT plan was smaller than the TW and FIF plan (p<0.05), but the difference of CI among the three inverse plans had no statistical significant. With respect to the HI of PTV, FIF and 7-IMRT plan had similar value of T-IMRT (p>0.05) between any two, but TW and VMAT plans were significantly worse (p<0.05). Figure 2 shows the dose distribution of five plans in isocentral slice.

### Left lung

Table 3 shows the D_mean_ and V_d_ of the left lung in different treatment techniques. The test results showed that the T-IMRT plan reduced the average dose and dose-volume except V_40_ compared with other plans. V_40_ of the three inverse plans were lower than TW and FIF plans significantly (p<0.05). However, the average V_40_ between any two of the three inverse plans had no statistical difference (p>0.05).

**Table 3:** **Dose comparison of the PRV-left lung between the five plans**$\left(\bar{x}\pm d\right)$

**Parameters**	**TW**	**FIF**	**T-IMRT**	**7-IMRT**	**VMAT**
D_mean_(Gy)	8.6±2.6^A^	8.2±2.4^A^	6.8±2.0^Ba^	9.3±3.8^A^	10.1±2.5^Bb^
V_5_ (%)	25.9±6.6^A^	24.6±6.1^A^	23.4±5.6^A^	49.4±9.5^B^	50.3±13.3^B^
V_10_ (%)	20.9±5.9^A^	19.1±5.5^A^	17.7±4.9^A^	26.8±6.2^B^	29.9±8.0^B^
V_20_ (%)	16.9±5.4^Ab^	15.0±5.0^A^	12.9±4.2^Aa^	14.6±4.5^A^	16.4±4.8^Ab^
V_30_ (%)	14.2±5.1^B^	12.2±4.6^A^	9.6±3.9^A^	9.6±3.9^A^	10.3±3.4^A^
V_40_ (%)	10.9±4.6^A^	8.7±3.9^A^	6.3±3.2^B^	4.7±2.5^B^	5.1±2.4^B^

A had significant difference with B (p<0.05), and, a had significant difference with b (p<0.05). Otherwise the difference was not significant between any two (p>0.05).

### Heart and coronary arteries

For the whole heart area, the average mean dose and V_5_~V_20_ of T-IMRT plan were smaller than all other plans significantly except FIF plan (P<0.05). The average V_30_ and V_40_ of three inverse plans were smaller than TW and FIF plans (P<0.05), and the difference between any two of the three inverse plans was similar (p>0.05).

The inverse plans also reduced the average mean dose and the V_10_~V_40_ of the coronary arteries compared to other two plans (p<0.05), and the difference of the three inverse plans was not statistical significant between any two (p>0.05). The V_5_ of T-IMRT plan was the smallest among the five plans (p<0.05).

Details of dose difference in PRV-heart and PRV-coronary artery were listed in Tables 4 and 5.

**Table 4:** **Comparison of the PRV-heart dose parameters in five plans**$\left(\bar{x}\pm d\right)$

**Parameters**	**TW**	**FIF**	**T-IMRT**	**7-IMRT**	**VMAT**
D_mean_(Gy)	3.7±2.0^Ab^	3.2±1.9^A^	2.2±1.0^Aa^	4.4±1.9^Ab^	4.6±1.7^Ab^
V_5_ (%)	10.2±6.0^A^	8.9±5.9^A^	6.3±3.6^A^	26.2±21.1^B^	26.1±15.1^B^
V_10_ (%)	7.5±5.0^Aa^	6.1±4.8^A^	3.5±2.4^Ab^	6.8±5.4^Aa^	6.9±4.9^Aa^
V_20_ (%)	5.6±4.2^A^	4.3±4.0^A^	2.0±1.7^Bb^	2.1±2.1^Bb^	2.5±2.4^Ba^
V_30_ (%)	4.2±3.5^A^	3.2±3.3^A^	1.2±1.3^B^	1.0±1.3^B^	1.1±1.5^B^
V_40_ (%)	3.0±2.7^A^	2.0±2.2^A^	0.6±0.9^B^	0.3±0.7^B^	0.4±1.0^B^

A had significant difference with B (p<0.05), and, a had significant difference with b (p<0.05). Otherwise the difference was not significant between any two (p>0.05).

**Table 5:** **Planned dose of PRV-coronary artery in five plans**$\left(\bar{x}\pm d\right)$

**Parameters**	**TW**	**FIF**	**T-IMRT**	**7-IMRT**	**VMAT**
D_mean_(Gy)	19.4±10.9^A^	15.5±10.2^A^	8.9±5.2^B^	9.9±4.7^B^	11.0±4.6^B^
V_5_ (%)	63.9±26.9^A^	56.8±26.8^Aa^	46.2±21.9^B^	66.2±28.2^A^	82.0±23.3^Ab^
V_10_ (%)	52.0±28.3^A^	42.4±24.6^Ba^	25.6±17.8^Bb^	28.6±18.3^Bb^	35.2±20.7^B^
V_20_ (%)	40.9±27.1^A^	31.2±26.6^A^	12.4±14.6^B^	11.9±14.5^B^	12.9±14.3^B^
V_30_ (%)	32.2±25.5^A^	23.5±25.0^A^	6.4±10.6^B^	5.5±9.9^B^	4.5±8.7^B^
V_40_ (%)	22.3±33.0^A^	14.3±19.9^A^	2.6±5.9^B^	1.3±4.0^B^	1.5±6.2^B^

A had significant difference with B (p<0.05), and, a had significant difference with b (p<0.05). Otherwise the difference was not significant between any two (p>0.05).

### Contralateral breast

The planned dose parameters of PRV-contralateral breast were listed in Table 6. It could be found that the average mean dose and V_2_~V_4_ of three tangential plans were lower than the other two plans (p<0.05), and the difference between any two of the three tangential plans was not statistical significant. The V_5_ of VMAT plan was the maximum of all five plans (p<0.05) and the difference of other four plans was not statistical significant. The V_10_ of five plans were all very small and were not significantly different between any two.

**Table 6:** **The dose parameters of PRV-coronary artery of five plans**$\left(\bar{x}\pm d\right)$

**Parameters**	**TW**	**FIF**	**T-IMRT**	**7-IMRT**	**VMAT**
D_mean_(Gy)	0.4±0.4^A^	0.4±0.4^A^	0.4±0.3^A^	1.6±0.7^B^	1.9±1.0^B^
V_2_ (%)	1.7±3.6^A^	2.2±3.9^A^	1.5±2.9^A^	29.4±21.2^B^	33.1±29.9^B^
V_3_ (%)	0.6±1.9^A^	0.6±2.0^A^	0.2±0.6^A^	13.9±15.2^B^	15.2±22.1^B^
V_4_ (%)	0.4±1.5^A^	0.4±1.6^A^	0.1±0.3^A^	5.8±8.1^Ba^	7.5±13.0^Bb^
V_5_ (%)	0.3±1.3^A^	0.3±1.4^A^	0.0±0.2^A^	1.6±3.2^A^	4.0±7.5^B^
V_10_ (%)	0.2±0.9^A^	0.2±0.9^A^	0.0±0.0^A^	0.0±0.0^A^	0.0±0.0^A^

A had significant difference with B (p<0.05), and, a had significant difference with b (p<0.05). Otherwise the difference was not significant between any two (p>0.05).

## Discussion

There have been many reports about the choice of radiation treatment technique for breast cancer after conserving surgery. Even in Rongsriyam’s report [21], T-IMRT should be the best treatment. However, Bhanagar A.K et al [22] found that the size of primary breast significantly affect the scatter dose to the contra-lateral breast. The PTV size of Chinese patients was smaller compared to those of the Caucasians. The mean and maximum size of PTV in our study was nearly one fourth to one third of those reported by Popescu CC. et al [10]. With respect to the dose parameters of PTV in this study, T-IMRT plan had obvious advantages on the HI than TW plan and VMAT plan, but it was not superior to the FIF plan and 7-IMRT plan when strict limitation was applied to the CI. In addition, T-IMRT plan had worse CI than 7-IMRT. This was different from the reported results of Jagsi et al [23], which might be the influence of the PTV size between European and Chinese. The multi-field plan and VMAT plan reduced the high dose-volume of PRV-OARs but increased the low dose-volume, and the VMAT plan even could not meet the constraint of PTV95%≥95%. To better conclude the most superior technique from the multi-parameter results of our study, we use the following score table to help making the evaluation. In the score table, it is scored to point 1 if the difference showed significant advance between the compared parameters, otherwise scored to 0. Thus, the best treatment technique goes to the one having highest score in the Table 7.

**Table 7:** Score table of the five treatment techniques

**Structure**	**Dose parameter**	**Treatment technique**				
		**TW**	**FIF**	**TIMRT**	**7IMRT**	**VMAT**
PTV	HI	1	1	1	1	0
	CI	0	0		1	1
	V_47.5_	1	0	1	1	0
PRV-l- lung	D_mean_	0		1	0	0
	V_5_	1	1	1	0	0
	V_10_	1	1	1	0	0
	V_20_	0	0	1	0	0
	V_30_	0	1	1	1	1
	V_40_	0	0	1	1	
PRV-Heart	D_mean_	0	1	1	0	0
	V_5_	1	1	1	0	0
	V_10_	0		1	0	0
	V_20_	0	0	1	1	1
	V_30_	0	0	1	1	1
	V_40_	0	0	1	1	1
PRV-coronary artery	D_mean_	0	0	1	1	1
	V_5_	0		1	0	0
	V_10_	0		1	1	1
	V_20_	0	0	1	1	1
	V_30_	0	0	1	1	1
	V_40_	0	0	1	1	1
PRV-r-breast	D_mean_	1	1	1	0	0
	V_2_	1	1	1	0	0
	V_3_	1	1	1	0	0
	V_4_	1	1	1	0	0
	V_5_	1	1	1	0	0
	V_10_	1	1	1	1	1
Score		10	12	26	14	12

The blank scoring: one who cannot be judged as superior nor inferior since it was not significant differ from both (p>0.05).

From the summary of scoring, T-IMRT has the most point of 26 which is almost 2 time of each all other technique. Although the PTV size was much smaller in this study, the score table led to similar result with the reported study of western cases. Caudell JJ.et al [24] reported that electronic compensation (CE) technique produced superior dose distribution in both CTV and normal tissue compared with conventional T-IMRT. One can expect that the dose distribution could be even better if CE technique was integrated.

**Figure 3:**
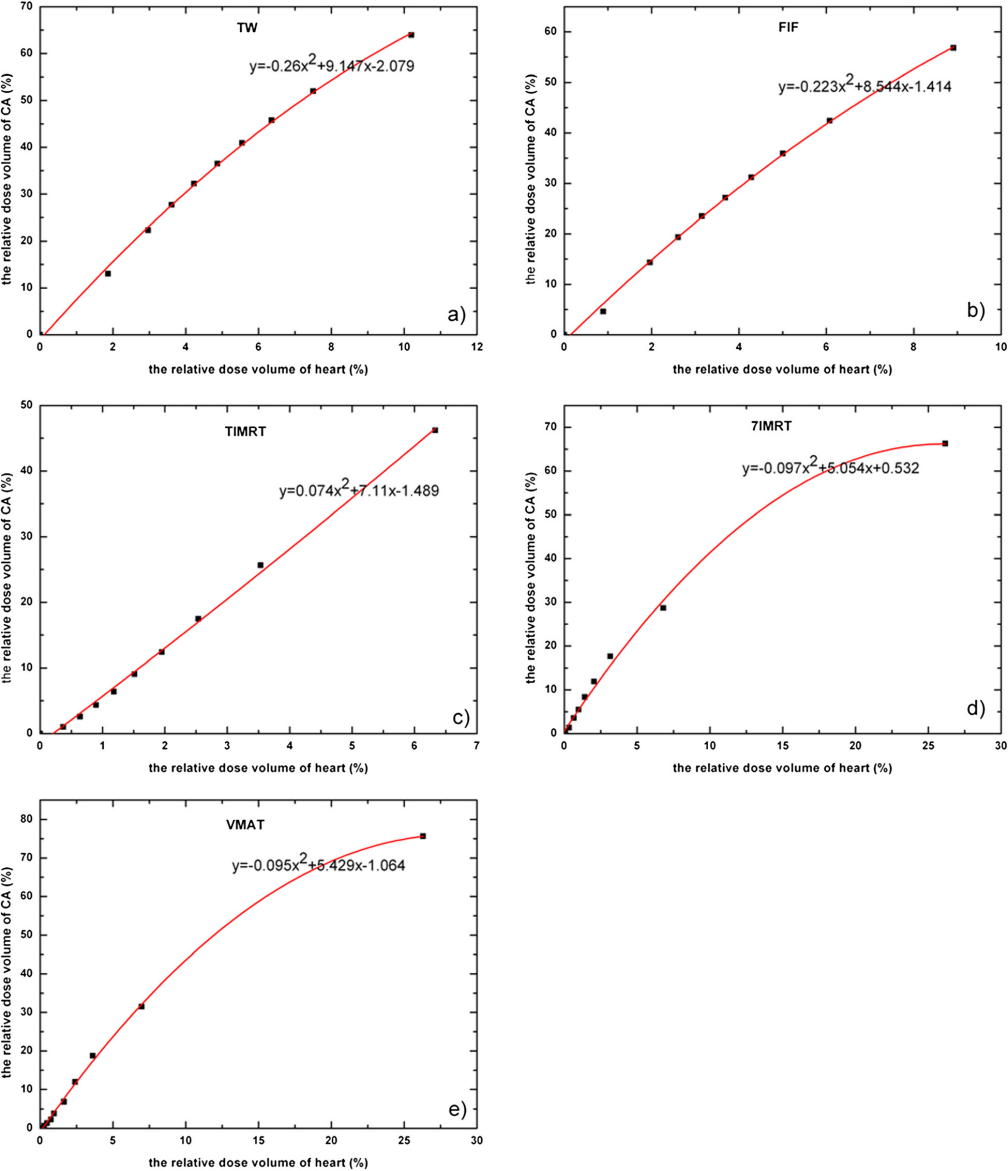
**The relationship between in dose volume of heart and coronary artery.** The abscissa and ordinate respectively represent the dose volume of heart and coronary artery. The red curve is the fitting curve.

The application of IMRT offers the potential for improved local-regional control without increase heart toxicity in those requiring local-regional treatments [25]. Darby SC et al. reported that exposure of the heart to ionizing radiation during radiotherapy for breast cancer increases the subsequent rate of ischemic heart disease [26]. Most of the literature analyzed the irradiated dose of heart, but they did not specify the dosimetric parameters of coronary artery when comparing the dose difference of treatment plans for the left-side breast cancer after conserving surgery. There were some studies suggested that coronary heart disease after postoperative radiotherapy for breast cancer was one of the radiation-related complications [27,28]. In this study, the dose-volume (V_5Gy_~V_40Gy_) of heart and coronary artery were detailed and their relationships were described with the quadratic polynomial. Figure 3 shows respectively the dose volume correlation of heart and coronary artery of the five plans. The correlation coefficients were 0.993 (TW), 0.996 (FIF), 0.9972 (T-IMRT), 0.995 (7-IMRT) and 0.986 (VMAT). This could be helpful to use the dose-volume of heart to predict the dose-volume of coronary artery. For example, if the V_30Gy_ of heart is 10% in T-IMRT, the V_30Gy_ of coronary artery can be calculated as 76.41% with the relation coefficient in Figure 3c). Dose-volume evaluation of coronary artery can be included in the constraint of heart dose. This was similar to the findings of other reports [29], which suggested to predict the dose of CA but not figured out the relationship in between.

Various reports have shown that the incidence of second cancer risk would increase with the increasing of the irradiated dose of contralateral breast [3]. Although 7-IMRT and VMAT were planned with same dose constraint for contralateral breast as the other techniques, the V_2_~V_5_ of contralateral breast were significant higher than the other three plans.

There are some reports about the application of VMAT in the clinical treatment of intact breast treatment with or without nodal involvement as well as for partial breast treatment [16]. In our study, the average MU of VMAT plan (363.7±45.3) was significantly smaller (P<0.05) than that of 7-IMRT (513.4±83.3MU). VMAT technique was superior in the irradiation MUs compared to 7-IMRT. Also, VMAT plan had apparent advantages in reducing the volume of high dose and drawbacks in increasing the volume of lower dose.

In our study, Comparing to the three tangential treatments VMAT reduced the normal tissue volume receiving high dose but significantly increased the volume of low dose. Especially, the average PTV95% of the 20 cases was worse than 95% when planned with same optimization objective of other treatment techniques. Considering the risk of tumor recurrence and the relative high dose in lung and contralateral breast, we do not suggest to choose VMAT for left-sided breast cancer radiation therapy.

## Conclusion

According to the data of our study, for the breast cancer patient whose PTV is rather smaller than western population, the size of primary breast do not significantly increase the dose of contra-lateral breast as reported by Bhanagar [22], and T-IMRT is still an adequate technique for the Chinese patients who undergo conserving breast surgery. For planning for left-breast irradiation, the volumetric dose of the heart which is more easy to be contoured can be used to predict the volumetric dose of coronary artery, if the relationship in between is well fitted. VMAT plan had a few advantage in improving the HI of PTV but may decrease the PTV dose coverage and increase the dose irradiate to lung and contra-lateral breast. The T-IMRT plan may be clinically.

## Consent

Written information consent was obtained from the patient for publication of this report and any accompanying images.

## Competing interests

The authors declare that they have no competing interests.

## Authors’ contributions

LXC is lead author, who designed the study and gave some advice on the manuscript revision. GHJ participated in data collection, data analysis, manuscript drafting, table/figure creation and manuscript revision. XWD and XWL gave some helpful advice about the study and the revision. YH and XBH gave some advice on the drawing of the target volume and OARs. All authors read and approved the final manuscript.
